# Incidence of hepatitis B virus infection among human immunodeficiency virus-infected treatment naïve adults in Botswana

**DOI:** 10.1097/MD.0000000000019341

**Published:** 2020-02-28

**Authors:** Bonolo Bonita Phinius, Motswedi Anderson, Resego Bokete, Tshepiso Mbangiwa, Wonderful Tatenda Choga, Kabo Baruti, Joseph Makhema, Rosemary Musonda, Jason T. Blackard, Max Essex, Sikhulile Moyo, Richard Marlink, Simani Gaseitsiwe

**Affiliations:** a Botswana Harvard AIDS Institute Partnership; b University of Botswana, Gaborone, Botswana; c Division of Immunology, Department of Pathology, University of Cape Town, South Africa; d Harvard T.H. Chan School of Public Health AIDS Initiative, Boston, Massachusetts; e University of Cincinnati College of Medicine, Cincinnati, Ohio; f Rutgers University, New Jersey, USA.

**Keywords:** Botswana, hepatitis B virus incidence, hepatitis B virus, human immunodeficiency virus, Sub-Saharan Africa

## Abstract

Hepatitis B virus (HBV) and human immunodeficiency virus (HIV) coinfection is highest in sub-Saharan Africa and results in accelerated clinical outcomes compared with HBV or HIV mono-infection. HBV clearance rates are higher in healthy adults; however, in sub-Saharan Africa, there are limited data on clearance of incident HBV in HIV-infected adults. Therefore, we sought to estimate HBV incidence and HBV surface antigen (HBsAg) clearance in HIV-infected adults in Botswana.

This was a retrospective longitudinal study of 442 HIV-1C infected treatment naïve patients enrolled in a previous Botswana Harvard AIDS Institute Partnership study. Archived plasma samples from 435 HIV-infected treatment naïve participants were screened for HBsAg and HBV core antibody (anti-HBc). HBsAg was evaluated annually over a 4-year period, and HBV deoxyribonucleic acid (DNA) levels of HBsAg-positive chronic and incident patients were quantified.

Baseline median CD4+ T-cell count was 458 cells/μL [Q1, Q3: 373, 593], and median HIV viral load was 4.15 copies/mL [Q1, Q3: 3.46, 4.64]. Twenty two HBV incident cases occurred, representing an incidence of 3.6/100 person-years [95% CI: 2.2–5.6]. All incident HBV cases with a follow-up sample available for screening (13/22) cleared HBsAg. Detectable HBV viral loads among chronic and incident cases ranged between 5.15 × 10^1^ to 1.4 × 10^7^ IU/L and 1.80 × 10^1^ to 1.7 × 10^8^ IU/mL, respectively.

We report high HBV incidence associated with elevated HBV DNA levels despite high CD4+ T-cell counts in HIV-infected patients in Botswana. These incidence cases represent a potential source of HBV transmission in the population. Scaling-up of HIV treatment strategies utilizing antiretroviral therapy regimens with anti-HBV activity coupled with screening for HBV infections in households of the HBsAg-positive cases is recommended.

## 1 Introduction

Hepatitis B virus (HBV) infection is a major health problem causing ∼887,000 deaths worldwide in 2015, with most of these deaths resulting from chronic liver disease.^[1]^ Sub-Saharan Africa is among the regions with the highest HBV-associated mortality.^[2]^ People infected with both human immunodeficiency virus (HIV) and HBV are at an increased risk of morbidity and mortality compared with those infected with either virus alone.^[3–6]^ Sub-Saharan Africa accounts for 70% of the global burden of HBV/HIV coinfection.^[7]^ The prevalence of HBV varies in distinct risk populations, and this has been shown by our previous studies in Botswana. For instance, the HBV prevalence—as determined by HBV surface antigen (HBsAg) positivity—among blood donors is 1.02%^[8]^ and ranges from 3.1% to 10.6% among HIV-infected individuals in Botswana.^[8–13]^ Coinfection of HBV with HIV increases the chance of higher chronicity rates and a general worse off clinical outcome than with either mono-infection.^[14]^ Several studies have also reported HBsAg clearance in HBV/HIV coinfected treatment experienced patients^[13,15,16]^; however, data on HBsAg clearance in HBV/HIV coinfected patients who are treatment naïve are limited.

Botswana is burdened with a high HIV-1 subtype C (HIV-1C) prevalence of 22.8% among adults between the ages of 15-49 years^[17]^; hence, the urgency to address issues of HBV infection in HIV-positive individuals. In a population based study in Botswana 83.3% of individuals knew their HIV status, and of these 87.4% were receiving antiretroviral therapy (ART) while 96.5% of all who were on ART were virologically suppressed.^[18]^ It is therefore important to evaluate factors that may hinder this success and also evaluate current efforts in the reduction of HBV transmissions in HIV-infected patients regardless of treatment status. Currently, there are no data on the incidence rate of HBV in HIV-infected individuals in Botswana. This study aimed to determine the incidence of HBV in HIV-infected treatment naïve patients in Botswana and investigate the risk factors associated with incident HBV infection.

## 2 Methods

### 2.1 Study participants

This was a retrospective longitudinal study of 442 HIV-1C infected treatment naïve patients enrolled in a previous Botswana Harvard AIDS Institute Partnership (BHP) study called Botsogo: The Natural HIV-1 Subtype C Disease Progression. Botsogo enrolled 442 treatment naïve adults with CD4^+^ T cell counts of >400 cells/μL and no AIDS-defining illness.^[19]^ The aim of this study was to observe HIV disease progression among these participants between the years 2005 and 2009^[19]^ and was approved by the Health Research Development Committee at the Botswana Ministry of Health and Wellness and the Office of Human Research Administration at the Harvard T. H. Chan School of Public Health. All participants gave informed consent.

### 2.2 Hepatitis B virus serological screening

All available plasma samples were serologically screened for HBsAg using the Murex Version 3 (Diasorin, Dartford, UK) enzyme linked immunosorbent assay (ELISA) kit, per the manufacturer's instructions at 4 time points that were ∼12 months apart when samples were available. All HBsAg-positive samples were confirmed by retesting. To assess previous exposure, the HBV core antibody (anti-HBc) was screened in all available samples (296) at the second time point (year 1) using the Monolisa anti-HBc PLUS ELISA kit (Bio-Rad, Paris, France). Chronic HBV cases were defined as ≥2 consecutive HBsAg-positive results while incident HBV cases are those with a HBsAg-positive result following a HBsAg-negative result.

### 2.3 HBV DNA quantification

Samples with confirmed chronic HBV infection and incident HBV cases were tested for HBV DNA using the COBAS AmpliPrep/COBAS TaqMan HBV Test v 2.0 (Roche Diagnostics, Mannheim, Germany) with a lower limit of quantification of 20 IU/mL. For all incident cases, HBV DNA was tested at the point of confirmed HBV incidence. For chronic HBV infections, this was performed at the earliest time point with an available plasma sample.

### 2.4 Statistical analysis

Statistical analysis was performed for 242 participants that had all available demographic data. Participants with missing data were excluded from analysis. Baseline participant characteristics were presented as proportions and medians. Incident cases and uninfected patients were compared using Fisher exact test for categorical data and the Wilcoxon rank sum test for continuous variables. We estimated HBV incidence with 95% confidence interval (CI) where incidence was reported in person-time. Follow-up time for each patient was calculated from the baseline date of enrolment to the exact visit date of the first HBsAg result for HBsAg-positive cases and to the last date of an available sample for those that remained HBsAg-negative. Cox proportional regression method was used to estimate hazard ratios (sex, age, CD4+ T cell count [≤450 or >450] cells/μL as prior studies have suggested a cut-off of 450 cells/μL for ART initiation for an increased survival rate as compared with lower cell counts.^[20,21]^ HIV viral load suppression [≤400 or >400] copies/mL) was also assessed as previously described.^[22,23]^*P* values <.05 were considered statistically significant. Non-invasive methods were used to determine the level of liver damage included aspartate aminotransferase (AST), alanine aminotransferase (ALT), AST platelet ratio index (APRI), and the Fibrosis 4 index.^[24]^ Liver damage was compared between the uninfected and incident cases using 2 sample Wilcoxon rank-sum (Mann–Whitney *U* test). The upper limit of normal (ULN) for ALT and AST were considered to be 42 and 41 U/L respectively. The demographics and some results were retrieved from BHP database. CD4+ T cells counts were measured using the BD FACSCalibur platform (BD Biosciences, San Jose, CA). Plasma HIV RNA levels were measured using COBAS AmpliPrep/COBAS AMPLICOR HIV-1 MONITOR Test, version 1.5 (Roche Molecular Systems, Branchburg, NJ). AST and ALT were measured using COBAS Integra plus (Roche Diagnostics, Rotkreuz, Switzerland). HBV testing for all participants was done prior separations into the different categories and groups to reduce any chance of biasness.

## 3 Results

**Figure 1:**
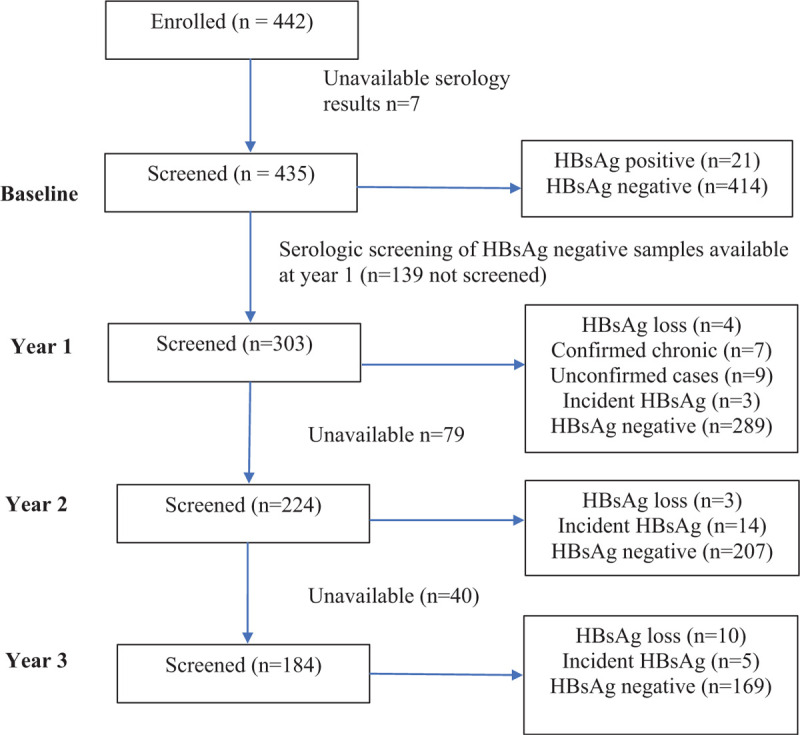
Participant screening algorithm over a 4-year period. HBsAg = Hepatitis B surface antigen.

**Figure 2:**
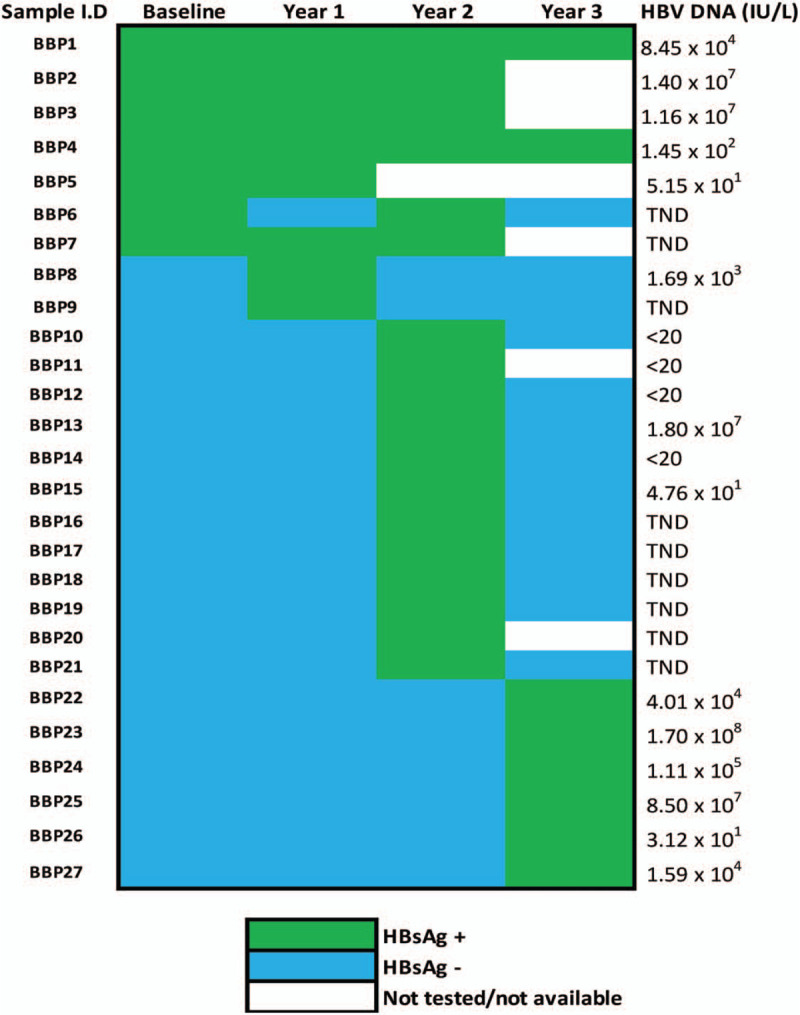
HBsAg screening for chronic and incident cases tested for HBV viral load, DNA = deoxyribonucleic acid, HBV = Hepatitis B virus, TND = Target not detectable, Chronic HBV—BBP1 to BBP7, Incident HBV- BBP8 to BBP27.

The Botsogo study recruited 442 participants, of which 435 were screened for HBsAg at baseline (Fig. 1). Twenty-one participants were HBsAg+ representing a prevalence of 4.8% (95% CI 3.01–7.16). Four of the 21 patients (19%) lost HBsAg at year 1, while 7 (33.3%) were considered chronic HBV infections as they were HBsAg+ for >6 months. However, for 9 (42.9%) patients, chronicity or lack thereof was not confirmed as subsequent samples were unavailable for screening. One patient (BBP6) showed an interesting trend in HBsAg status: HBsAg+ at baseline, antigen clearance at year 1 only to be positive again for the surface antigen at year 2, followed by a loss of the antigen at year 3 (Fig. 2). At year 1, there were 3 HBsAg+ cases that were HBsAg^−^ at baseline (i.e., incident cases). At year 2, 224 samples were screened including year 1 incident cases. All year 1 incident cases had lost HBsAg at this time point. A total of 14 incident cases were observed, of which all 10 that had available samples at the subsequent time point cleared HBsAg. In the last time point (year 3), 5 incident cases (HBsAg^+^) were observed (Fig. 1). Of the 296 participants tested for anti-HBc at year 1, 133 (44.9%) were anti-HBc positive at year 1. Two of these patients only tested positive for the HBsAg at year 2 (data not shown).

### 3.1 HBV viral load

Seven chronic HBV patients (>6 months since infection) and 20 incident HBV patients were tested for HBV DNA (Fig. 2). HBV DNA levels among chronic and incident cases ranged from 5.15 × 10^1^ to 1.4 × 10^7^ IU/mL and 1.80 × 10^1^ to 1.7 × 10^8^ IU/mL, respectively. Two of 7 (28.6%) chronic HBV and 7 of 20 (35%) incident HBV infections had a “target not detectable” (TND) result. In addition, 5 of 20 (25%) incident HBV infections had DNA levels <20 IU/mL.

The median age of the participants was 32 (interquartile range [IQR]: 28, 39), and 81.4% of the participants were women. The median baseline CD4+ T cell count was 458 cells/μL (IQR: 373,593), and the median baseline log HIV viral load was 4.15 (IQR: 3.46, 4.64). All participants had normal liver enzyme levels and normal platelet count at baseline. There were no statistically significant differences between HBV uninfected and incident cases based on APRI, FIB4, AST, ALT, CD4^+^ T cell count, sex, anti-HBc, or HIV viral load. The median age of incident HBV participants was 28 (IQR: 27, 31), while that of uninfected participants was 32 (IQR: 28, 40; *P* value = .0330). (Table 1). HBV incident cases were higher in patients that had attended secondary school (*P* value = .045) (Table 1).

**Table 1:**
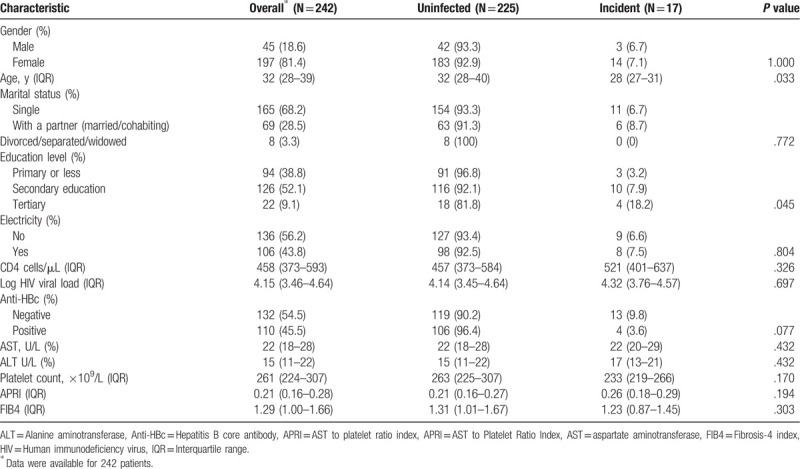
Association of baseline demographics and HBV incidence.

There were no statistically significant differences in HBV incidence based on sex, age, CD4^+^ T cell count, or HIV viral load. Participants who were 35 years or younger were approximately 4 times more likely to have incident HBV; however, as with other factors, this was not statistically significant (Table 2).

**Table 2:**
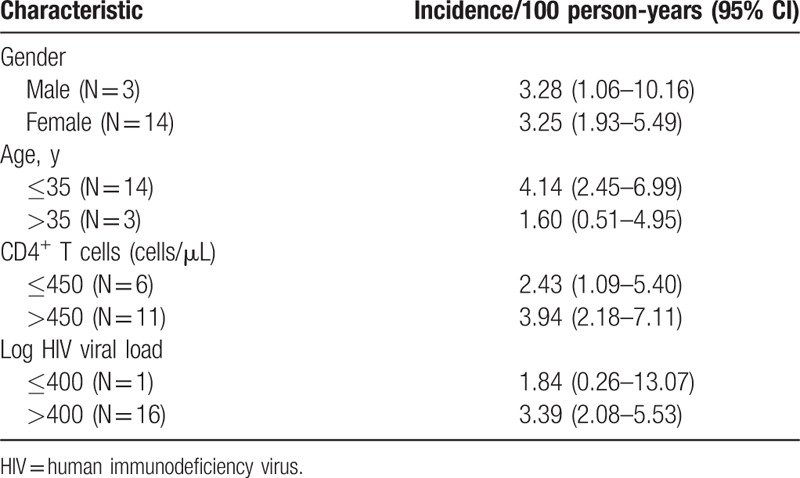
Factors associated with incident HBV.

### 3.2 HBV incidence and risk factors

**Figure 3:**
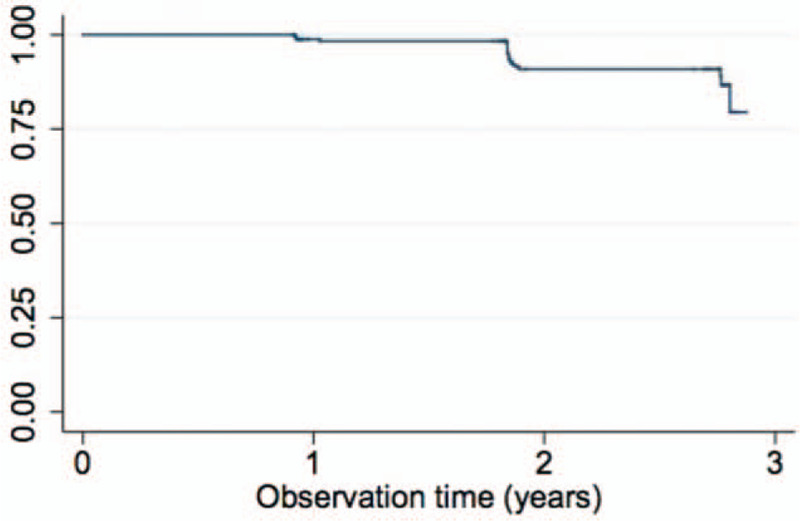
Kaplan–Meier curve for proportion of HBV survival. HBV = Hepatitis B virus.

Participants contributed 620.35 person-years to the study, and they were followed for a median of 2.75 years (IQR: 1.82–2.76). Cumulatively, there were 22 incident cases, giving an incidence of 3.6 (95% CI, 2.3–5.4) per 100 person-years. All incident cases with available follow-up samples were negative for HBsAg at the next time point (i.e., they cleared HBsAg). Approximately 75% of the followed-up population did not get infected with HBV over 3 years of follow-up. Median time to incident HBV was 673 days (IQR, 672–681) (Fig. 3).

## 4 Discussion

This is the first study to determine HBV incidence in Botswana. The baseline prevalence of HBsAg was similar to rates reported in previous studies in Botswana among HIV-positive participants.^[10,11,13,25]^ It should be noted that confirmed chronic HBV infections constituted 7 of 21 individuals who were HBsAg+ at baseline, hence the need to interpret the prevalence with caution to avoid an overestimation of chronic HBV in this population. We observed a high HBsAg clearance rate in this cohort after years of follow-up, which has not been addressed previously by other studies.

There was high HBsAg clearance in this study. It is known that immune-competent adults that get infected with HBV in adulthood tend to clear the HBsAg at a high rate of between 90% and 95%^[26]^ and the participants of this study were no exception despite the fact that they were HIV-infected. There are limited data on HBsAg clearance rates in HBV incident cases; however, a study in Rome reported an HBsAg clearance rate of about 82% in acute HBV patients.^[27]^ According to our knowledge, in Botswana, HBsAg clearance has only been reported for chronic HBV patients where 37% of participants on anti-HBV treatment (tenofovir and emtricitabine) over a 2-year period cleared HBsAg.^[13]^

Several mechanisms may facilitate the clearance of HBV during acute infection, including cytokines released by HBV-specific T cells at the site of infection.^[28]^ Stelma et al^[26]^ observed functional HBV-specific CD8^+^ T cell and CD4^+^ T cell responses in patients who cleared HBV and lower responses in patients who developed chronic HBV infections. Viral factors have also been shown to be associated with HBsAg loss such as genotype A and D,^[29]^ which could likely be the case in our study as these genotypes are also common in Botswana.^[9,30]^

Our estimated incidence of 3.6 per 100 person-years in HIV-infected treatment naïve patients is higher than a previous report in a similar population in Uganda, where the incidence was found to be 2.3/100 person-years.^[31]^ The HBV incidence rate for HIV-infected patients on ART was 0.49/100 person-years in Uganda and 4.2/1000 person-years in Rwanda,^[31,32]^ far lower than our estimated incidence rate. However, there are limited data on the incidence of HBV in HIV-infected treatment naïve adults in sub Saharan Africa. Other HBV incidence studies in Africa have been done in blood donor groups and have shown lower incidents rates in those populations^[33,34]^; however, higher incidence rates were observed in blood donors in Cote d’Ivoire.^[35]^

We observed 2 patients with an uncommon phenomenon; they showed a positive anti-HBc result at year 1, however, with a negative HBsAg result that seroconverted to HBsAg+ after 12 months. Further tests to evaluate possible occult HBV infections (HBsAg–negative but HBV DNA positive) were not performed. Various explanations could be attributed to these 2 cases. We postulate that these could be cases of reactivation as positive anti-HBc results indicate previous exposure to HBV. Previously, cases of reactivation in patients with HBc-positivity have been described in patients of different clinical histories, with interrupted lamivudine treatment,^[36–38]^ and without antiretroviral therapy interruption.^[39]^ One other patient's HBsAg status switched between positive and negative results at each time points throughout follow-up and may be attributed to reactivation.

There was a statistically significant difference between incident cases and participants who did not get infected with age. This finding has also been observed in Uganda.^[31]^ However, it is to be noted that none of the risk factors evaluated had a statistical significance in the risk of acquiring incident HBV. This is in contrast to a study in Uganda that showed that younger people and those with an HIV viral load >400 copies/mL had a higher risk of being infected throughout the duration of the study.^[31]^

There is a need to further assess the impact of socioeconomic factors on HBV infection in Botswana. In our study, more incident cases were observed in participants with secondary education than in primary or tertiary education. This is in contrast to a study of pregnant women in Tanzania where a higher HBsAg prevalence was found in women with primary education^[40]^; however, a study in Nigeria had similar findings to our findings.^[41]^ In a similar study in Kenya, there was no significant difference in HBV prevalence according to education level.^[42]^ The level of knowledge on HBV may be crucial in that individuals will be aware of vaccination and treatment options, as well as prevention strategies.

We report HBV inactive carriers in our cohort characterized by HBsAg positivity and very low to undetectable HBV DNA levels who are at a risk of reactivation as shown in other studies.^[43,44]^ This has also been observed in a previous study of patients initiating ART in Botswana.^[13]^ We also observe high viral loads in incident HBV patients who later cleared the surface antigen. High HBsAg seroclearance has been observed in patients with high HBV viral load at baseline after treatment with tenofovir disoproxil fumarate (TDF).^[45]^ However, these findings require further investigations. Despite the high HBV seroclearance, implications for the incident HBV infections associated with high HBV viral loads is a cause for concern and warrants consideration for antiretroviral regimens with anti-HBV active drugs.

The clinical implications of the current findings are diverse. All incident cases with an available sample to be screened at a consecutive time point cleared the surface antigen, hence being labeled as acute HBV (AHB). In Italy, AHB is of clinical concern as it most often requires hospital admission sometimes being a threat to the patient's life. It was related to strains from elsewhere,^[27]^ and this raised concern of treatment of immigrants. Furthermore, Jindal et al^[46]^ state that reactivation of HBV is associated with immunosuppression and will most often present as AHB, hence the need to further assess predictors of chronicity in these patients, particularly because they are coinfected with HIV. Other populations such as pregnant women also need to be assessed for incidence of HBV because of their delayed clearance of the surface antigen hence pregnancy being a possible risk of chronicity.^[47]^ Our results are not generalizable to the HIV negative patients or the HIV positive patients that are on ART, therefore assessments in these cohorts are necessary.

In conclusion, we observe high incidence of HBV in HIV-infected adults compared with other studies in different parts of sub-Saharan Africa. This raises a clinical concern because these patients were generally healthier with higher CD4^+^ T cell counts and without AIDS-defining illnesses. As Botswana has adopted a Treat All strategy, it is even more vital to evaluate this achievement and combat all major possible hindrances to the immune restoration of affected individuals. As such, early screening of HBV in HIV-infected individuals is vital and should be in national HIV treatment guidelines. Furthermore, screening HBV in households of the HBsAg positive cases is recommended as HBV can be transmitted horizontally.

This study bears the strength of longitudinal follow-up of incident cases and therefore the ability to identify them as acute infections. A limitation to the study was the lack of a control group of HIV uninfected patients and HIV-infected treatment experienced patients particularly in a Treat All era. There is also a possibility that some HBV incident cases might have been missed as screenings were 12 months apart which is a long interval. Further work to determine incidence of occult HBV in this population is necessary as the use of serology only has been shown to underestimate HBV prevalence through missing out occult infections.^[48]^ We estimated HBV incidence from all participants including those that tested positive to anti-HBc which may be an overestimation posing another limitation of our study in addition to the high numbers of loss to follow up and unavailable samples. As previously discussed, we have 2 confirmed cases of reactivation in the study indicated. It should be noted that out of the 22 incident cases, 3 were had a positive anti-HBc result.

The main findings of this study are the high HBV incidence rate 3.6 per 100 person-years and clearance rate.

## Acknowledgments

The authors would like to acknowledge the participants of the Botsogo study. They would also like to acknowledge the Botswana Harvard AIDS Institute Research Laboratory for their generous support.

## Author contributions

BBP, SG, SM, MA conceptualized the study and designed the experiments. BBP, RB, WTG, KB conducted the experiments. BBP and SM analysed the results. BBP and MA wrote the manuscripts. JTB provided expert review. SG, SM, TM, ME, RMM, RM and JM reviewed, edited and approved the manuscript.

Bonolo Bonita Phinius orcid: 0000-0002-0180-2705.

Motswedi Anderson orcid: 0000-0001-9974-9684.

Resego Bokete orcid: N/A.

Tshepiso Mbangiwa orcid: N/A.

Wonderful Tatenda Choga orcid: 0000-0001-7606-0569.

Kabo Baruti orcid: N/A.

Joseph Makhema orcid: 0000-0003-0017-2438.

Rosemary Musonda orcid: 0000-0001-5028-1515.

Jadon T Blackard orcid: 0000-0003-2876-3811.

Max Essex orcid: N/A.

Sikhulile Moyo orcid: 0000-0003-3821-4592.

Richard Marlink orcid: N/A.

Simani Gaseitsiwe orcid: 0000-0002-7089-3735.

## References

[1] Kim SJ Jang JY Kim EJ Ginsenoside Rg3 restores Hepatitis C virus-induced aberrant mitochondrial dynamics and inhibits virus propagation. Hepatology 2017;66:758–71. 28329914 10.1002/hep.29177 PMC5755973

[2] Stanaway JD Flaxman AD Naghavi M The global burden of viral hepatitis from 1990 to 2013: findings from the Global Burden of Disease Study 2013. Lancet 2016;388:1081–8. 27394647 10.1016/S0140-6736(16)30579-7 PMC5100695

[3] Kourtis AP Bulterys M Hu DJ HIV-HBV coinfection--a global challenge. N Engl J Med 2012;366:1749–52. 22571198 10.1056/NEJMp1201796 PMC4453872

[4] Thio CL Seaberg EC Skolasky R HIV-1, hepatitis B virus, and risk of liver-related mortality in the Multicenter Cohort Study (MACS). Lancet 2002;360:1921–6. 12493258 10.1016/s0140-6736(02)11913-1

[5] Chun HM Mesner O Thio CL HIV outcomes in Hepatitis B virus coinfected individuals on HAART. J Acquir Immune Defic Syndr 2014;66:197–205. 24694929 10.1097/QAI.0000000000000142 PMC4034265

[6] Puglia M Stasi C Da Fre M Prevalence and characteristics of HIV/HBV and HIV/HCV coinfections in Tuscany. Braz J Infect Dis 2016;20:330–4. 26748234 10.1016/j.bjid.2015.11.007 PMC9427616

[7] Likis F Hepatitis C. J Midwifery Women Health 2017;62:243–4. 10.1111/jmwh.12623 28340501

[8] Choga WT Anderson M Zumbika E Molecular characterization of hepatitis B virus in blood donors in Botswana. Virus Genes 2018;55:33–42. 30382563 10.1007/s11262-018-1610-z PMC6563613

[9] Mbangiwa T Kasvosve I Anderson M Chronic and occult Hepatitis B virus infection in pregnant women in Botswana. Genes (Basel) 2018;9:E259. 29772814 10.3390/genes9050259 PMC5977199

[10] Khudyakov YE Matthews PC Beloukas A Prevalence and characteristics of Hepatitis B Virus (HBV) coinfection among HIV-positive women in South Africa and Botswana. PLoS One 2015;10:e0134037. 26218239 10.1371/journal.pone.0134037 PMC4517770

[11] Mandiwana A Tshitenge S Prevalence of human immunodeficiency virus — hepatitis B virus co-infection amongst adult patients in Mahalapye, Ngami, Serowe, Botswana: a descriptive cross-sectional study. South Afr Fam Pract 2017;59:94–7.

[12] Wester CW Bussmann H Moyo S Serological evidence of HIV-associated infection among HIV-1-infected adults in Botswana. Clin Infect Dis 2006;43:1612–5. 17109297 10.1086/508865

[13] Anderson M Gaseitsiwe S Moyo S Slow CD4(+) T-Cell recovery in human immunodeficiency virus/Hepatitis B virus-coinfected patients initiating Truvada-based combination antiretroviral therapy in Botswana. Open Forum Infect Dis 2016;3:ofw140. 27800524 10.1093/ofid/ofw140 PMC5084712

[14] Morikawa K Shimazaki T Takeda R Hepatitis B: progress in understanding chronicity, the innate immune response, and cccDNA protection. Ann Transl Med 2016;4:337. 27761441 10.21037/atm.2016.08.54 PMC5066049

[15] Hamers RL Zaaijer HL Wallis CL HIV-HBV coinfection in Southern Africa and the effect of lamivudine- versus tenofovir-containing cART on HBV outcomes. J Acquir Immune Defic Syndr 2013;64:174–82. 23892239 10.1097/QAI.0b013e3182a60f7d

[16] Nunez M Ramos B Diaz-Pollan B Virological outcome of chronic hepatitis B virus infection in HIV-coinfected patients receiving anti-HBV active antiretroviral therapy. AIDS Res Hum Retroviruses 2006;22:842–8. 16989608 10.1089/aid.2006.22.842

[17] UNAIDS. Botswana: HIV and AIDS estimates. In: Joint United Nations Programme on HIV/AIDS (UNAIDS); 2017.

[18] Gaolathe T Wirth KE Holme MP Botswana's progress toward achieving the 2020 UNAIDS 90-90-90 antiretroviral therapy and virological suppression goals: a population-based survey. Lancet HIV 2016;3:e221–30. 27126489 10.1016/S2352-3018(16)00037-0 PMC5146754

[19] Farahani M Novitsky V Wang R Prognostic Value of HIV-1 RNA on CD4 trajectories and disease progression among antiretroviral-Naive HIV-Infected Adults in Botswana: a joint modeling analysis. AIDS Res Hum Retroviruses 2016;32:573–8. 26830351 10.1089/aid.2015.0348 PMC4892219

[20] Ntekim AI Folasire AM CD4 count and anti retroviral therapy for HIV positive patients with cancer in Nigeria -A Pilot Study. Clin Med Insights Oncol 2010;4:61–6. 20703325 10.4137/cmo.s5028 PMC2918360

[21] Jain V Deeks SG When to start antiretroviral therapy. Curr HIV/AIDS Rep 2010;7:60–8. 20425559 10.1007/s11904-010-0044-6 PMC2856854

[22] Assoumou L Weiss L Piketty C A low HIV-DNA level in peripheral blood mononuclear cells at antiretroviral treatment interruption predicts a higher probability of maintaining viral control. AIDS 2015;29:2003–7. 26355572 10.1097/QAD.0000000000000734

[23] Stöhr W Fidler S McClure M Duration of HIV-1 viral suppression on cessation of antiretroviral therapy in primary infection correlates with time on therapy. PLoS One 2013;8:e78287. 24205183 10.1371/journal.pone.0078287 PMC3808338

[24] WHO. Guidelines for the Prevention, Care and Treatment of Persons with Chronic Hepatitis B Infection. Geneva; 2015. 26225396

[25] Patel P Tolle M Anabwani G Prevalence of Hepatitis B and Hepatitis C coinfections in an adult HIV centre population in Gaborone, Botswana. Am J Trop Med Hyg 2011;85:390–4. 21813864 10.4269/ajtmh.2011.10-0510 PMC3144842

[26] Stelma F Willemse SB Erken R Dynamics of the immune response in acute Hepatitis B infection. Open Forum Infect Dis 2017;4:ofx231. 29302605 10.1093/ofid/ofx231 PMC5739046

[27] Menzo S Minosse C Vincenti D Long-term follow-up of acute hepatitis b: new insights in its natural history and implications for antiviral treatment. Genes (Basel) 2018;9:E293. 29895748 10.3390/genes9060293 PMC6027296

[28] Ferrari C HBV and the immune response. Liver Int 2015;35: suppl: 121–8. 25529097 10.1111/liv.12749

[29] Marcellin P Buti M Krastev Z Kinetics of hepatitis B surface antigen loss in patients with HBeAg-positive chronic hepatitis B treated with tenofovir disoproxil fumarate. J Hepatol 2014;61:1228–37. 25046847 10.1016/j.jhep.2014.07.019 PMC5976831

[30] Anderson M Gaseitsiwe S Moyo S Molecular characterisation of hepatitis B virus in HIV-1 subtype C infected patients in Botswana. BMC Infect Dis 2015;15:335. 26268355 10.1186/s12879-015-1096-4 PMC4535680

[31] Seremba E Ssempijja V Kalibbala S Hepatitis B incidence and prevention with antiretroviral therapy among HIV-positive individuals in Uganda. Aids 2017;31:781–6. 28099188 10.1097/QAD.0000000000001399 PMC5380792

[32] Rusine J Ondoa P Asiimwe-Kateera B High seroprevalence of HBV and HCV infection in HIV-infected adults in Kigali, Rwanda. PLoS ONE 2013;8:e63303. 23717409 10.1371/journal.pone.0063303 PMC3661584

[33] Namululi BA Guerrieri C Dramaix MW Prévalence et incidence du VIH et de l’hépatite B chez les donneurs de sang et estimation du risque résiduel de transmission du virus VIH et du virus VHB par la transfusion sanguine. Une étude à l’hôpital provincial général de référence de Bukavu, République démocratique du Congo Revue d’Épidémiologie et de Santé Publique 2013;61:139–44. 10.1016/j.respe.2012.09.005 23498094

[34] Nagalo BM Bisseye C Sanou M Seroprevalence and incidence of transfusion-transmitted infectious diseases among blood donors from regional blood transfusion centres in Burkina Faso, West Africa. Trop Med Int Health 2012;17:247–53. 21988100 10.1111/j.1365-3156.2011.02902.x

[35] Seri B Minga A Gabillard D Twenty-year evolution of Hepatitis B Virus and Human immunodeficiency virus prevalence and incidence in Voluntary blood donors in Cote d’Ivoire. Open Forum Infect Dis 2018;5:ofy060. 29644251 10.1093/ofid/ofy060 PMC5888498

[36] Chamorro AJ Casado JL Bellido D Reactivation of hepatitis B in an HIV-infected patient with antibodies against hepatitis B core antigen as the only serological marker. Eur J Clin Microbiol Infect Dis 2005;24:492–4. 15990987 10.1007/s10096-005-1355-1

[37] Bagaglio S Porrino L Lazzarin A Molecular characterization of occult and overt hepatitis B (HBV) infection in an HIV-infected person with reactivation of HBV after antiretroviral treatment interruption. Infection 2010;38:417–21. 20533073 10.1007/s15010-010-0032-1

[38] Costantini A Marinelli K Biagioni G Molecular analysis of hepatitis B virus (HBV) in an HIV co-infected patient with reactivation of occult HBV infection following discontinuation of lamivudine-including antiretroviral therapy. BMC Infect Dis 2011;11:310. 22054111 10.1186/1471-2334-11-310 PMC3239326

[39] Bani-Sadr F Maillard A Ponscarme D Reactivation of HBV replication in HIV-HBV infected patients. Am J Med 2003;114:768–9. 10.1016/s0002-9343(03)00156-6 12829207

[40] Manyahi J Msigwa Y Mhimbira F High sero-prevalence of hepatitis B virus and human immunodeficiency virus infections among pregnant women attending antenatal clinic at Temeke municipal health facilities, Dar es Salaam, Tanzania: a cross sectional study. BMC Pregnancy Childbirth 2017;17:109. 28388879 10.1186/s12884-017-1299-3 PMC5383970

[41] Anaedobe CG Fowotade A Omoruyi CE Prevalence, sociodemographic features and risk factors of Hepatitis B virus infection among pregnant women in Southwestern Nigeria. Pan Afr Med J 2015;20:406. 26301010 10.11604/pamj.2015.20.406.6206 PMC4524914

[42] Ngaira JA Kimotho J Mirigi I Prevalence, awareness and risk factors associated with Hepatitis B infection among pregnant women attending the antenatal clinic at Mbagathi District Hospital in Nairobi, Kenya. Pan Afr Med J 2016;24:315. 28154670 10.11604/pamj.2016.24.315.9255 PMC5267875

[43] Chung SJ Kim JK Park MC Reactivation of hepatitis B viral infection in inactive HBsAg carriers following anti-tumor necrosis factor-alpha therapy. J Rheumatol 2009;36:2416–20. 19797507 10.3899/jrheum.081324

[44] Holmes JA Yu ML Chung RT Hepatitis B reactivation during or after direct acting antiviral therapy - implication for susceptible individuals. Expert Opin Drug Saf 2017;16:651–72. 28471314 10.1080/14740338.2017.1325869 PMC5589072

[45] Gordon SC Krastev Z Horban A Efficacy of tenofovir disoproxil fumarate at 240 weeks in patients with chronic hepatitis B with high baseline viral load. Hepatology 2013;58:505–13. 23364953 10.1002/hep.26277 PMC3842114

[46] Jindal A Kumar M Sarin SK Management of acute hepatitis B and reactivation of hepatitis B. Liver Int 2013;33: suppl: 164–75. 23286861 10.1111/liv.12081

[47] Han YT Sun C Liu CX Clinical features and outcome of acute hepatitis B in pregnancy. BMC Infect Dis 2014;14:368. 24993389 10.1186/1471-2334-14-368 PMC4096733

[48] Ryan K Anderson M Gyurova I High rates of occult hepatitis b virus infection in hiv-positive individuals initiating antiretroviral therapy in Botswana. Open Forum Infect Dis 2017;4:ofx195. 29062862 10.1093/ofid/ofx195 PMC5641381

